# Supplementary material for: Cocaine-context memories are transcriptionally encoded in nucleus accumbens Arc ensembles
Source: Nat Commun. 2025 Jul 2;16:6084. doi: 10.1038/s41467-025-61004-9 (PMC12222677; doi:10.1038/s41467-025-61004-9)
Supplement: Supplementary file 1 — Supplementary Information [file 41467_2025_61004_MOESM1_ESM.docx]

**Supplementary Information**

Cocaine-context memories are transcriptionally encoded in nucleus accumbens Arc ensembles.

**Supplementary Figures**

**
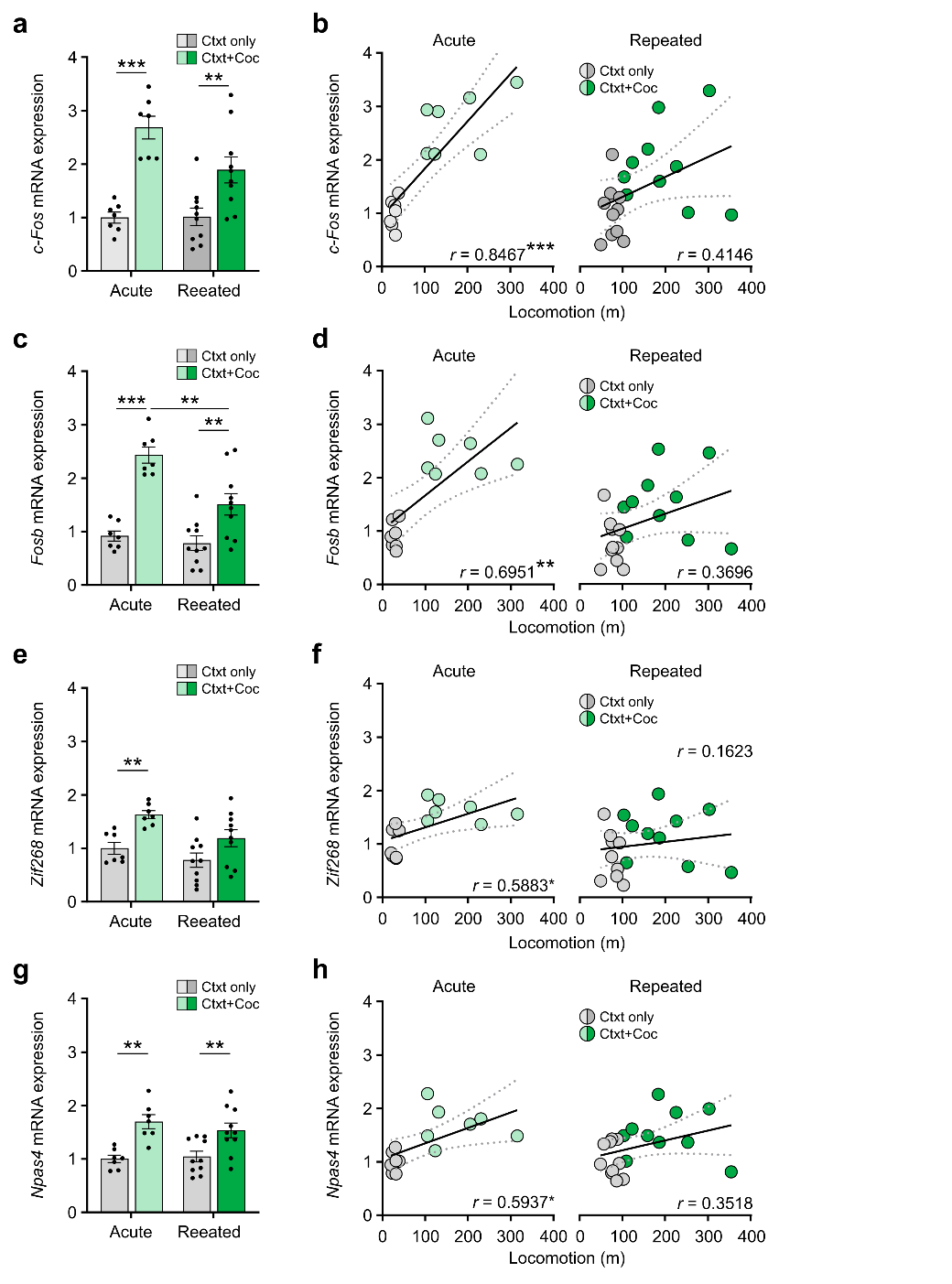
**

**Supplementary Figure 1: Expression of other immediate early genes after acute vs. repeated cocaine exposure and locomotor sensitization.** Expression is from the same tissue and experimental design as in Fig. 1 (n = 7, acute groups; n=10, repeated groups). **a**, Expression of *Fos* mRNA. Two-way ANOVA: interaction regimen x drug, F_1,30_ = 4.032; *p* = 0.0537; main effect of regimen F_1,30_ = 3.715, *p* = 0.0634; main effect of drug F_1,30_ = 40.27, ****p* < 0.0001; followed by Šidák post hoc tests. **b**, Correlation of *Fos* mRNA levels with locomotor activity. Acute (left): Pearson’s r = 0.8467, ****p* = 0.0001. Repeated (right): Pearson’s r = 0.4146, ***p* = 0.0691. **c**, Expression of *Fosb* mRNA. Two-way ANOVA: interaction regimen x drug, F_1,30_ = 5.704, **p* = 0.0234; main effect of regimen F_1,30_ = 10.29, ***p* = 0.0032; main effect of drug F_1,30_ = 46.33, ****p* < 0.0001; followed by Šidák post hoc tests. **d**, Correlation of *Fosb* mRNA levels with locomotor activity. Acute (left): Pearson’s r = 0.6951, ***p* = 0.0058. Repeated (right): Pearson’s r = 0.3696, *p* = 0.1087. **e**, Expression of *Zif268* mRNA. Two-way ANOVA: interaction regimen x drug, F_1,30_ = 0.6596, *p* = 0.4231; main effect of regimen F_1,30_ = 5.996, **p* = 0.0204; main effect of drug F_1,30_ = 14.81, ****p* = 0.0006; followed by Šidák post hoc tests. **f**, Correlation of *Zif268* mRNA levels with locomotor activity. Acute (left): Pearson’s r = 0.5883, **p* = 0.0269. Repeated (right):Pearson’s r = 0.1623, *p* = 0.4941. **g**, Expression of *Npas4* mRNA. Two-way ANOVA: interaction regimen x drug, F_1,30_ = 0.7516, *p* = 0.3929; main effect of regimen F_1,30_ = 0.2569, *p* = 0.6159; main effect of drug F_1,30_ = 24.30, ****p* < 0.0001; followed by Šidák post hoc tests. **h**, Correlation of *Npas4* mRNA levels with locomotor activity. Acute (left):Pearson’s r = 0.5937, **p* = 0.0252. Repeated (right): Pearson’s r = 0.3518, *p* = 0.1282. Bar graphs are expressed as means ± SEM. Correlation graphs show the regression line with a 95% confidence interval. Source data are provided as a Source Data file.

**
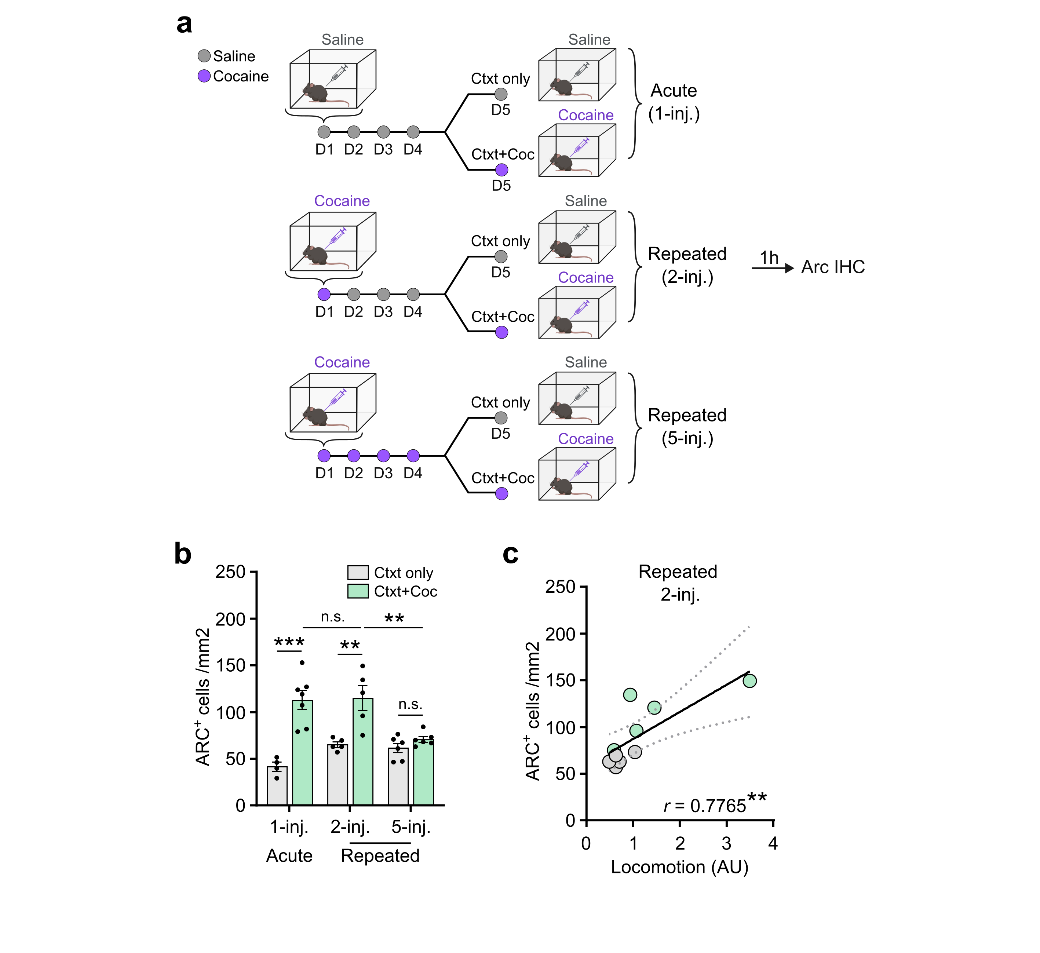
**

**Supplementary Figure 2: A single re-exposure to cocaine is not associated with a decrease in ARC ensemble size. a,** Experimental design for experimenter-administered cocaine regimens in C57BL/6J mice. Acute and repeated regimens are compared with a single cocaine re-exposure where animals are injected twice with cocaine 4 days apart. Mice were injected i.p. with cocaine (20 mg/kg) or saline in an open-field arena different from their home cage. On day 5, tissue was collected 1 h after the last injection. IHC, immunohistochemistry, Ctxt = context, Coc = cocaine, 1-inj = 1 injection, 2 inj. = 2 injections, 5-inj. = 5 injections. **b,** The repeated 2-inj. group showed an increased number of ARC+ cells (green, ARC+ ensemble) in NAc after cocaine as compared to saline, to a similar extent as the acute 1-inj. group. Cocaine-mediated ARC induction in the 2-inj. group was significantly higher than in the repeated 5-inj. group. n = 4, acute/ctxt only; n = 7, acute/ctxt+coc; n=5, 2-inj./ctxt only and 2-inj./ctxt+coc; n=6, 5-inj./ctxt only; n = 7, 5-inj./ctxt+coc. Two-way ANOVA: interaction regimen x drug, F_2,27_ = 8.266; ***p* = 0.0016; main effect of regimen F_2,27_ = 4.642, **p* = 0.0185; main effect of drug F_1,27_ = 44.88, ****p* < 0.0001; followed by Šidák post-hoc tests. **c,** The number of ARC+ cells correlated positively with cocaine-induced locomotion in the 2-inj. group. Pearson’s r = 0.7765, ***p* = 0.0083. Bar graphs are expressed as means ± SEM. Correlation graphs show the regression line with a 95% confidence interval. Source data are provided as a Source Data file.

**
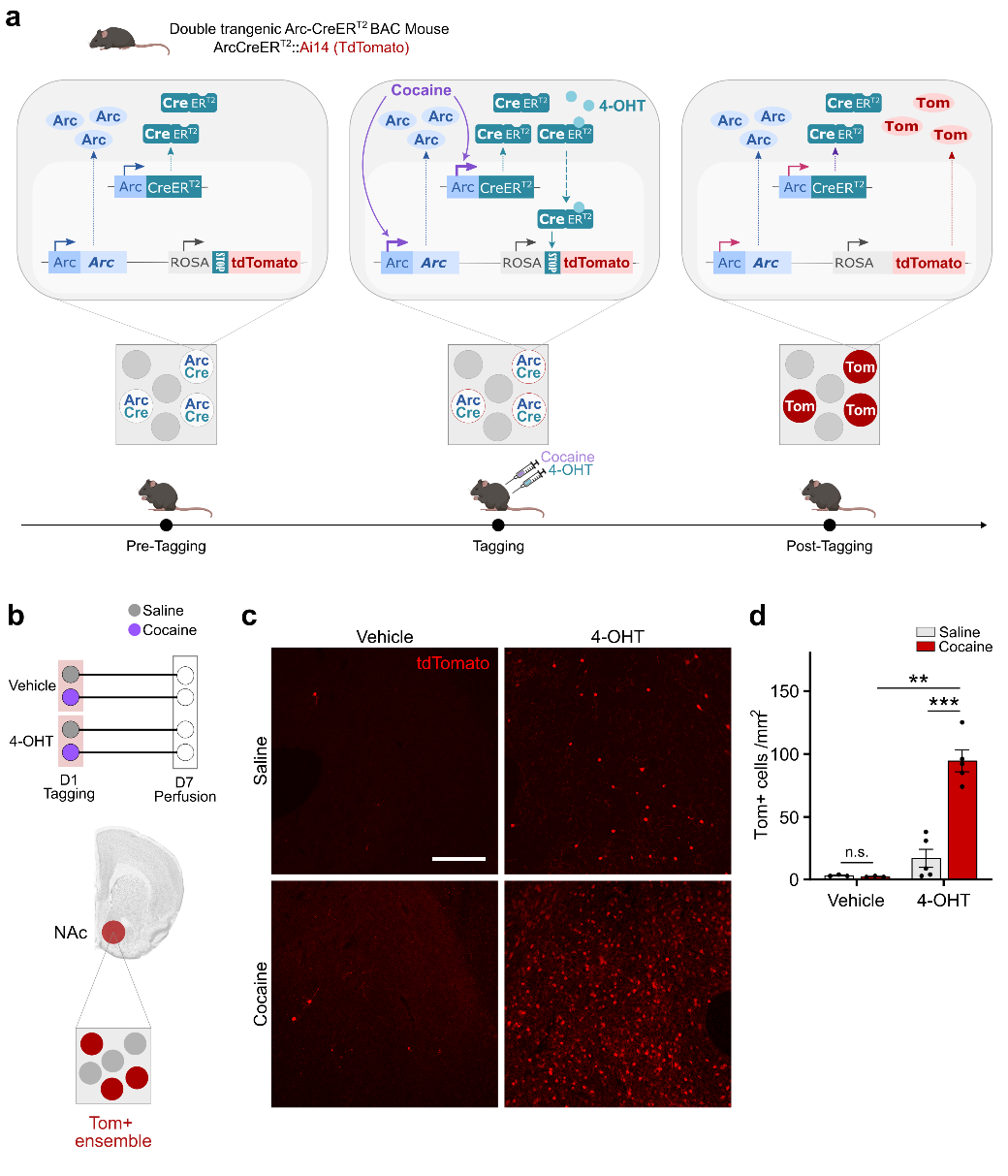
**

**Supplementary Figure 3**: **Permanent tagging of ARC+ ensembles in ArcCreER^T2^::Ai14 mice**. **a,** Schematic representation of the strategy applied to tagging cocaine-recruited ARC+ ensembles in NAc. ArcCreER^T2^ mice were crossed with the Ai14 reporter line to induce stable tdTomato expression in *Arc*-expressing cells. Ensemble tagging is achieved via the concomitant injection of 4-OHT and cocaine. Cocaine triggers induction of both endogenous ARC and the CreER^T2^ transgene. Upon 4-OHT binding, CreER^T2^ enters the nucleus where it removes the floxed-STOP cassette and enables tdTomato expression. The persistent expression of tdTomato in cocaine-activated cells allows for their visualization at any future time (e.g., 7 days). Tom, tdTomato; 4-OHT, 4-Hydroxytamoxifen. **b,** On day 1 mice were injected i.p. with cocaine (20 mg/kg) or saline along with 4-OHT (10 mg/kg) or vehicle in their home cage. Tissue was collected 7 days later to visualize the ensembles previously recruited by cocaine. **c,** Representative confocal images of Tom+ cells (red, Tom+ ensemble) in NAc. Scalebar, 100 µM. **d,** As compared to saline, cocaine increased the number of Tom+ cells in the 4-OHT-treated group, but not in the vehicle-treated one. n = 3, Vehicle; n = 5, 4-OHT. Two-way ANOVA: Interaction treatment x pre-treatment, F_1,12_ = 27.48, ***p = 0.0002; main effect of treatment, F_1,12_ = 26.09; ***p = 0.003; main effect of pre-treatment, F_1,12_ = 49.98, ***p < 0.0001; followed by Šidák post-hoc tests. Bar graphs are expressed as means ± SEM. Source data are provided as a Source Data file.

**
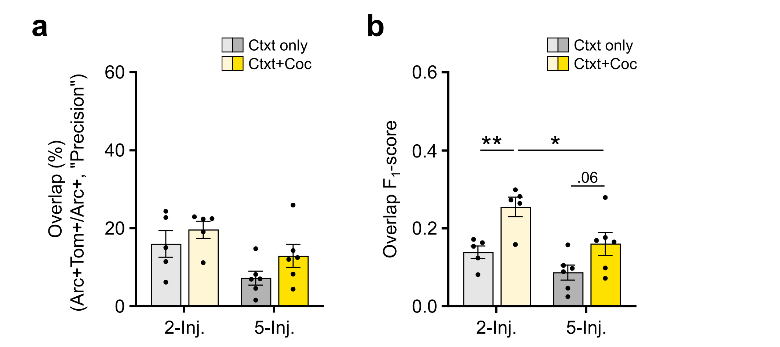
**

**Supplementary Figure 4: Additional ensemble overlap metrics in locomotor sensitization. a**, Overlap between ensembles activated by first and last cocaine injection, expressed as a “Precision” ratio over the total number of Arc+ cells at the last injection. Two-way ANOVA: interaction regimen x challenge, F_1,18_ = 0.15, *p* = 0.7014; main effect of regimen, F_1,18_ = 8.50, ***p* = 0.0092; main effect of challenge, F_1,18_ = 3.10, *p* = 0.0953; followed by Šidák post-hoc tests. **b**, Overlap between ensembles activated by first and last cocaine injection, expressed a F_1_-score (see Methods for details). Two-way ANOVA: interaction regimen x challenge, F_1,18_ = 0.83, *p* = 0.3747; main effect of regimen, F_1,18_ = 9.72, ***p* = 0.0059; main effect of challenge, F_1,18_ = 16.15, *p* = 0.0008; followed by Šidák post-hoc tests. Bar graphs are expressed as means ± SEM. Source data are provided as a Source Data file.

**
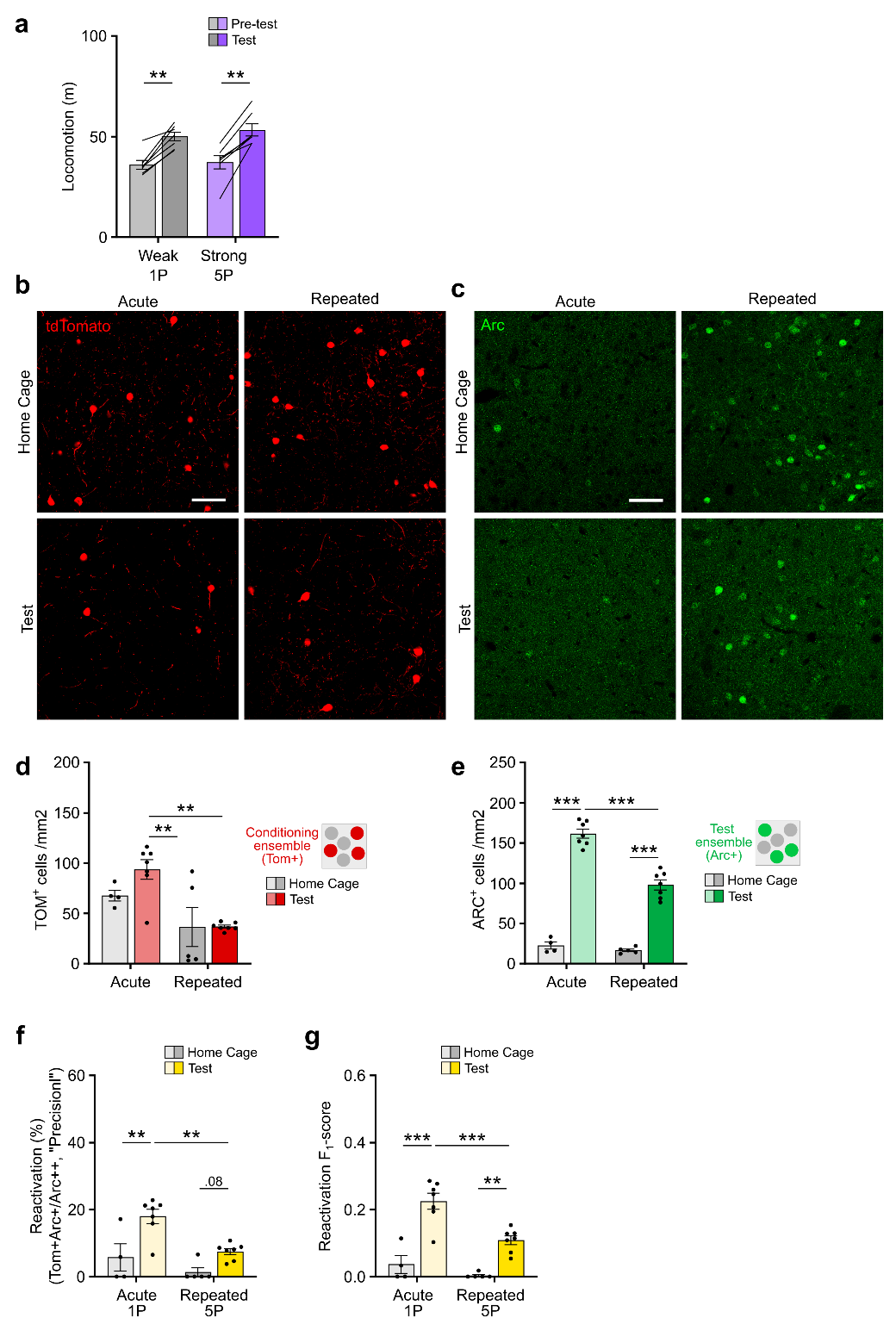
**

**Supplementary Figure 5: Recruitment of NAc ensembles during weak vs. strong conditioning and expression of cocaine-associated memories. a**, Locomotor activity during CPP testing before and after conditioning. Two-way RM-ANOVA: interaction conditioning x session F_1,24_ = 0.14, *p* = 0.7138; main effect of conditioning F_1,24_ = 0.71, *p* = 0.4082; main effect of session F_1,24_ = 30.88, ****p* < 0.0001; followed by Šidák post-hoc tests. **b,** Representative confocal images of the tdTomato (Tom+) cells (red, conditioning ensemble) that are recruited in NAc during the conditioned phase of the cocaine conditioned place preference (CPP) paradigm. **c,** Representative confocal images of the Arc+ cells (green, recall ensemble) that are recruited in NAc during the conditioned phase of the cocaine conditioned place preference (CPP) paradigm. **d,** IHC quantification showed a significantly higher number of cells recruited in the 1-pairing vs. 5-pairing groups without any effect of the test as compared to the home cage. n = 4, acute/home cage; n = 7, acute/test; n = 5, repeated/home cage; n = 7, repeated/test. Two-way ANOVA: interaction conditioning x test, F_1,19_ = 1.440, *p* = 0.2448; main effect of conditioning, F_1,19_ = 16.73, ****p* = 0.0006; main effect of test, F_1,19_ = 1.522, *p* = 0.2323; followed by Šidák post hoc tests. **d,** The number of ARC+ cells increased after the test session as compared to the home cage condition in both the 1-pairing and 5-pairing groups, with a significantly lower induction in the 5-pairing group. n = n = 4, acute/home cage; n = 7, acute/test; n = 5, repeated/home cage; n = 7, repeated/test. Two-way ANOVA: interaction conditioning x test, F_1,19_ = 26.29, ****p* < 0.0001; main effect of conditioning, F_1,19_ = 3851, ****p* < 0.0001; main effect of test, F_1,19_ = 3845, ****p* < 0.0001; followed by Šidák post hoc tests. **f**, Reactivation of the encoding (CPP conditioning) ensemble upon memory recall (CPP testing or home cage controls), expressed as a “Precision” ratio over the total number of Arc+ cells upon recall. Two-way ANOVA: interaction conditioning x test, F_1,19_ = 2.09, *p* = 0.1643; main effect of conditioning, F_1,19_ = 12.64, ***p* = 0.0021; main effect of test, F_1,19_ = 19.39, ****p* = 0.0003; followed by Šidák post-hoc tests. **g**, Reactivation between ensembles activated by CPP encoding and recall, expressed a F_1_-score (see Methods for details). Two-way ANOVA: interaction conditioning x test, F_1,19_ = 4.41, **p* = 0.0493; main effect of conditioning, F_1,19_ = 14.31, ***p* = 0.0013; main effect of test, F_1,19_ = 54.98, ****p* < 0.0001; followed by Šidák post-hoc tests. Bar graphs are expressed as means ± SEM. Source data are provided as a Source Data file.


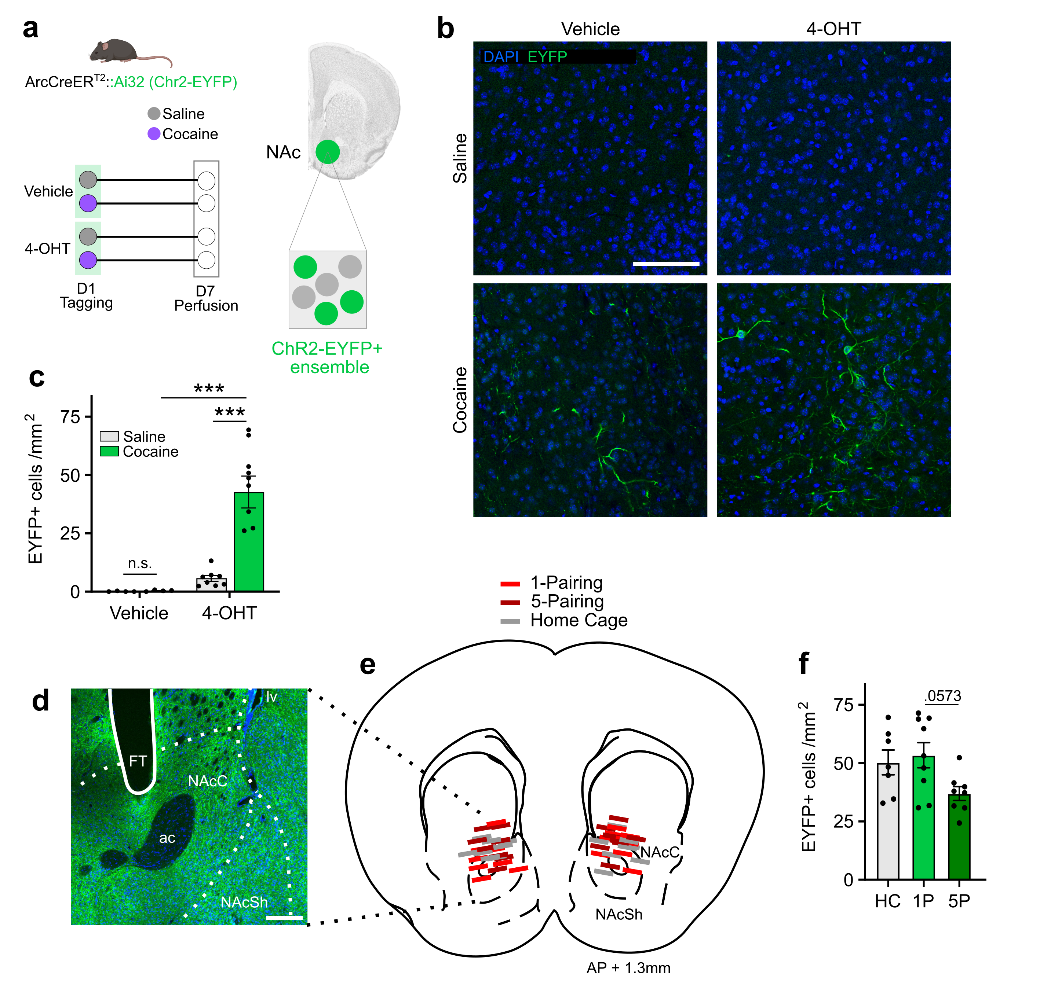


**Supplementary Figure 6**: **Permanent tagging of ARC+ ensembles in ArcCreER^T2^::Ai32 mice**. **a,** On day 1, mice were injected i.p. with cocaine (20 mg/kg) or saline along with 4-hydroxytamoxifen (4-OHT, 10 mg/kg) or vehicle in their home cage. Tissue was collected 7 days later to visualize the ensembles previously recruited by cocaine. **b,** Representative confocal images of EYFP+ cells (green, ChR2-EYFP+ ensemble) in NAc. Scalebar, 100 µM. **c,** As compared to saline, cocaine increased the number of EYFP+ cells in the 4-OHT-treated group, but not in the vehicle-treated group. n = 4, Vehicle; n = 8, 4-OHT/Saline, n = 6, 4-OHT/Cocaine. Two-way ANOVA: Interaction treatment x pre-treatment, F_1,18_ = 20.34, ***p = 0.0003; main effect of treatment, F_1,18_ = 21.04; ****p* = 0.0002; main effect of pre-treatment, F_1,18_ = 34.50 ***p < 0.0001. **d**, Representative fiber tract (FT) above NAc in a ArcCreER^T2^::Ai32 mouse with ChR2-EYFP expression. ac anterior commissure, lv lateral ventricle, NAcC NA core, NAcSh NAc shell. Scale bar 100µm. **e**, Schematic representation of fiber tract placements. **f**, Quantification of ChR2-EYFP+ tagged cells across pairing groups. HC home cage. n = 7-9. One-way ANOVA: F_2,21_ = 2.701, *p* = 0.0488; followed by Šidák post-hoc tests. Bar graphs are expressed as means ± SEM. Source data are provided as a Source Data file.

**
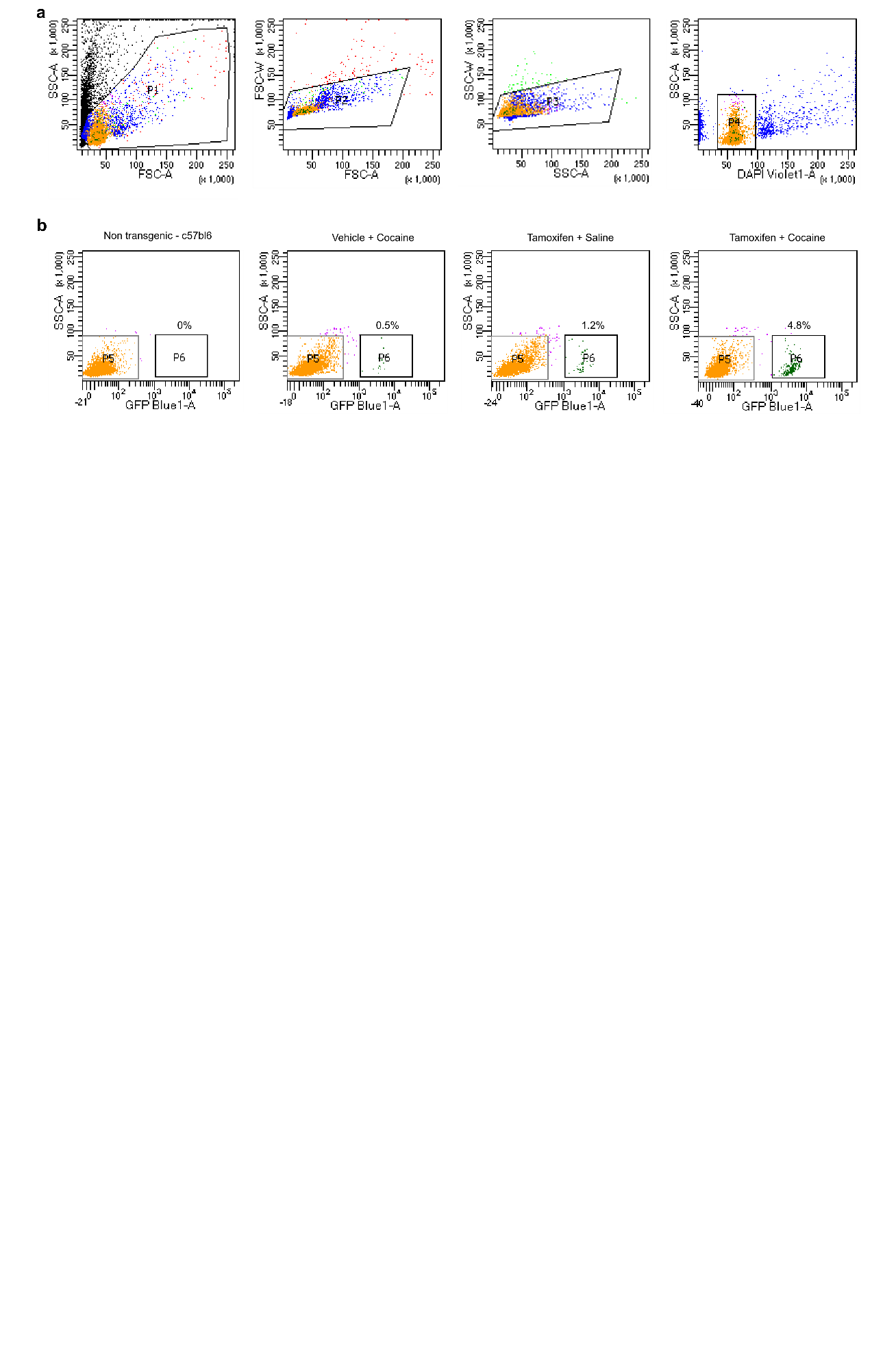
**

**Supplementary Figure 7: Fluorescence-Activated Nuclei Sorting (FANS) of striatal ARC neuronal ensembles. a,** Representative FANS gating strategy from a ArcCreERT2::Sun1 NAc sample. **b,** Visualization of FANS-isolated non-ensemble (GFP-) and ensemble (GFP+) nuclei and percent of GFP+ nuclei for non-transgenic sample or ArcCreERT2::Sun1 treated with a combination of 4-OHT (or vehicle) along with cocaine (or saline). 4-OHT (or vehicle as a control) was injected concomitantly with cocaine (or saline as a control) in the home cage and NAc collected 7 days later for nuclei isolation and FANS-based ensemble isolation. Source data are provided as a Source Data file.

**
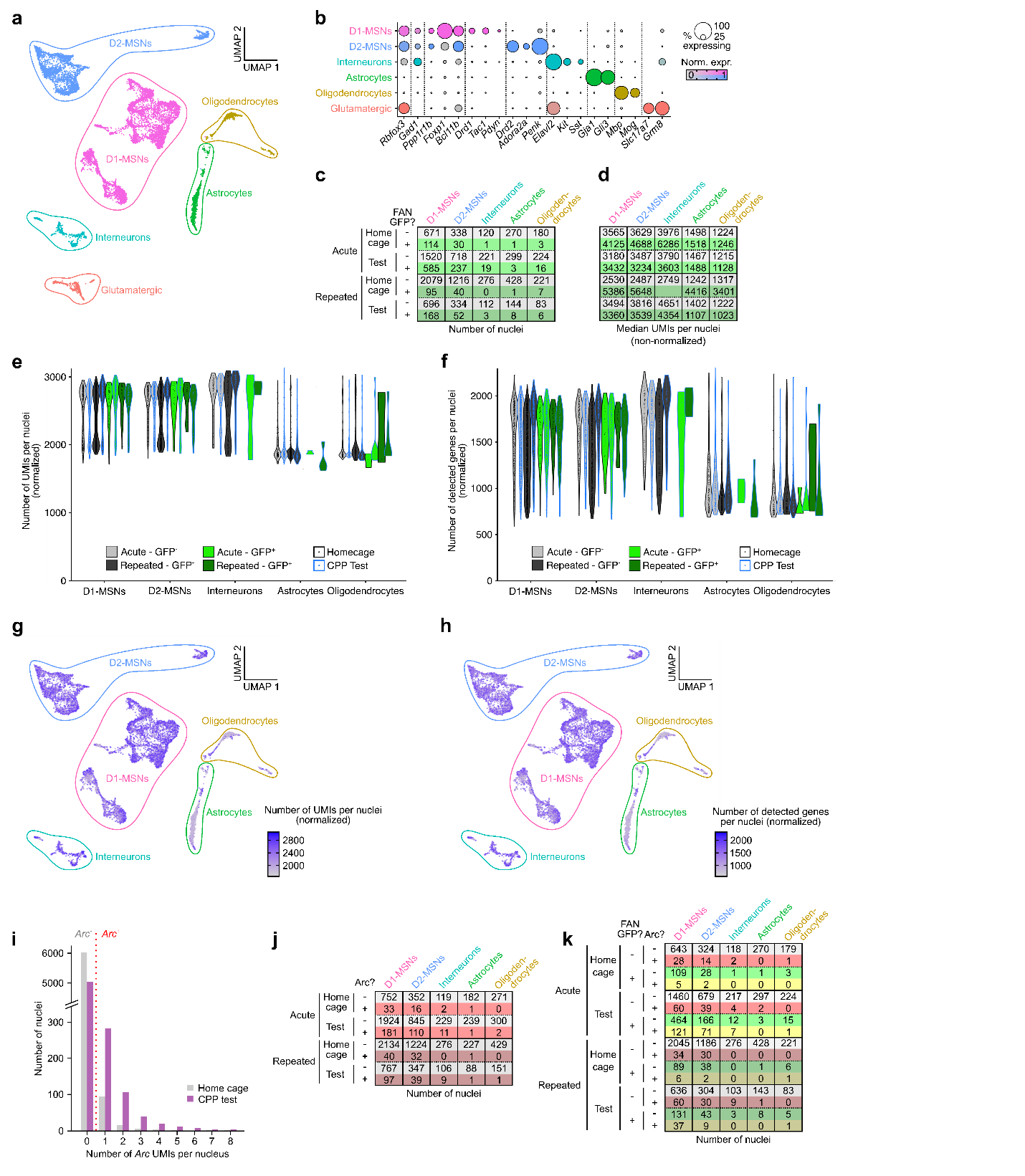
Supplementary Figure 8:** **Cell type annotation of snRNAseq clusters and quality control metrics.** **a,** UMAP reduction and cell-type annotation of all collected nuclei (n = 11,539), segregated into phenotypically defined clusters and colored according to their cluster of origin. **b,** Expression of published marker genes ^56,57^ for striatal cell types across clusters. Full list of cluster marker genes is given in Supplementary Information Table 2. **c,** Number of nuclei from each group and FANS status for each cell type. **d,** Median number of unique molecular identifiers (UMIs) detected in nuclei from each group and FANS status for each cell type before SCT normalization. **e**, Number of UMIs per nucleus after SCT normalization in each group and FANS status for each cell type as violin plot. **f**, Number of detected genes per nuclei after SCT normalization in each group and FANS status for each cell type as violin plot. **g**, Number of UMIs per nuclei after SCT normalization as UMAP reduction. **f**, Number of detected genes per nuclei after SCT normalization as UMAP reduction. **i**, Distribution of *Arc* UMIs. Dotted line indicates Arc^+^ threshold. **j,** Number of nuclei from each group and *Arc* status for each cell type. **k,** Number of nuclei from each group and FANS status and Arc status for each cell type. Source data are provided as a Source Data file.

**
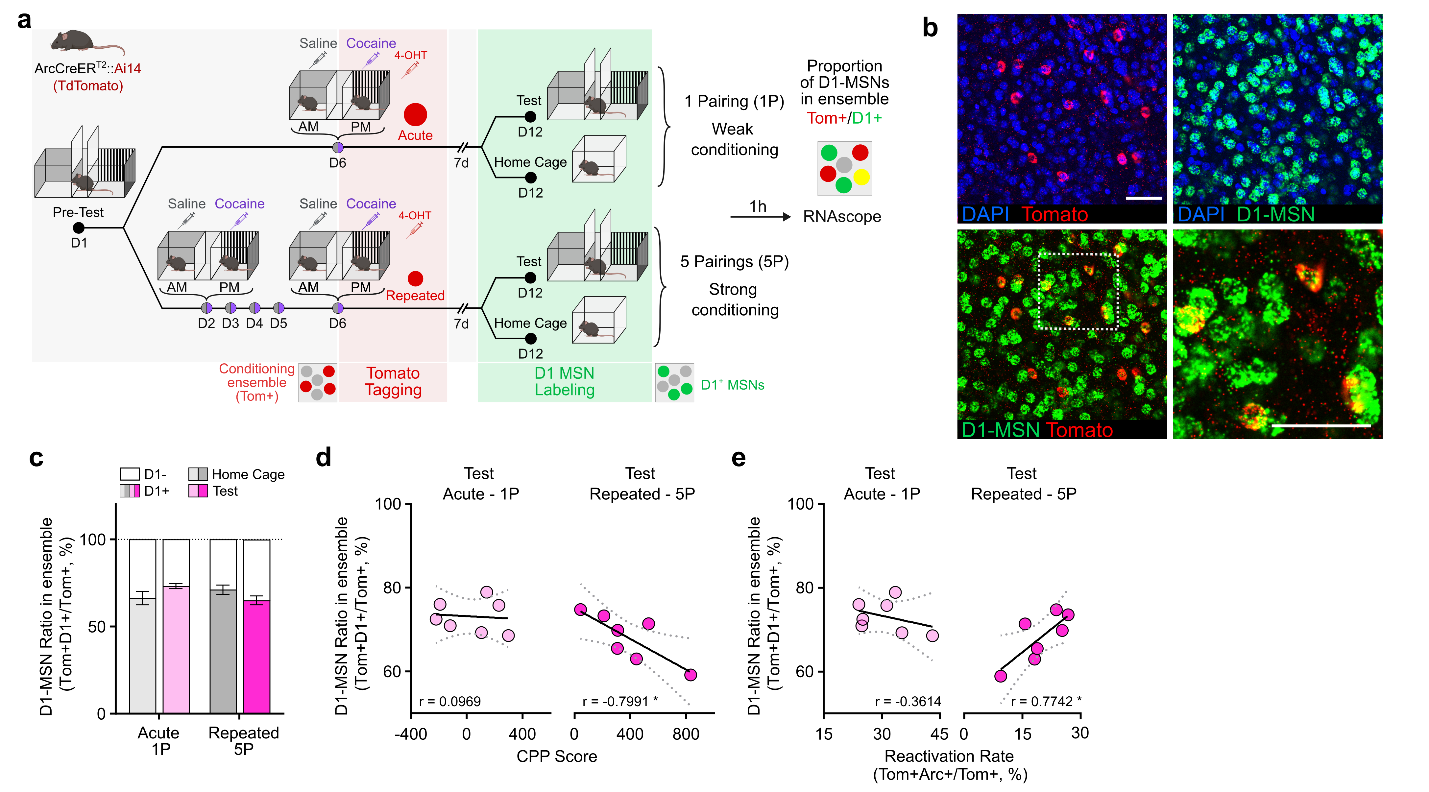
Supplementary Figure 9: Differential recruitment of D1 vs. D2 MSN populations in cocaine activated NAc ensembles.** **a,** Experimental design for ensemble tagging in ArcCreER^T2^::Ai14 mice in a cocaine conditioned place preference (CPP) protocol. Mice were conditioned with cocaine (20 mg/kg i.p.) on one day or on five consecutive days and injected i.p. with 4-hydroxytamoxifen (4-OHT, 10 mg/kg) at the beginning of the last conditioning session. Mice were then tested 7 days later for their preference for the cocaine-paired chamber. NAc tissue was collected 1 h after the beginning of the test session and processed for RNAscope. **b,** Representative confocal images of RNAScope staining showing the colocalization between Tom+ cells (red, Tom+ ensemble) activated during conditioning and D1+ MSNs. Scale bar, 50 µM. **c,** RNAScope quantification showed a similar proportion of D1 MSN among the acute and repeated ensembles. n = 4, 1P/home cage; n = 5, 5P/home cage; n= 7, 1P/test; n = 10, 5P/test. Two-way ANOVA: interaction conditioning x test, F_1,22_ = 5.372, **p* = 0.0302; main effect of conditioning, F_1,22_ = 03629, *p* = 0.5531; main effect of test, F_1,22_ = 0.0147, *p =* 0.9046; followed by Šidák post-hoc tests. **d,** The ratio of D1 MSNs in the ensemble correlated negatively with the CPP score in the 5-pairing group (right), but not the 1-pairing group (left). 1-pairing (left): n = 7; Pearson’s r = - 0.0969, *p* = 0.8363. 5-pairing (right): n = 7, Pearson’s r = - 0.7991, * *p* = 0.0311. **e,** The ratio of D1 MSNs in the ensemble showed significant positive correlation with ensemble reactivation in the 5-pairing group (right), but not the 1-pairing group (left). 1-pairing (left): n = 7; Pearson’s r = - 0.3614, *p* = 0.4257. 5-pairing (right): n = 7, Pearson’s r = 0.7742, * *p* = 0.0410. Bar graphs are expressed as means ± SEM. Correlation graphs show the regression line with a 95% confidence interval. Source data are provided as a Source Data file.

**
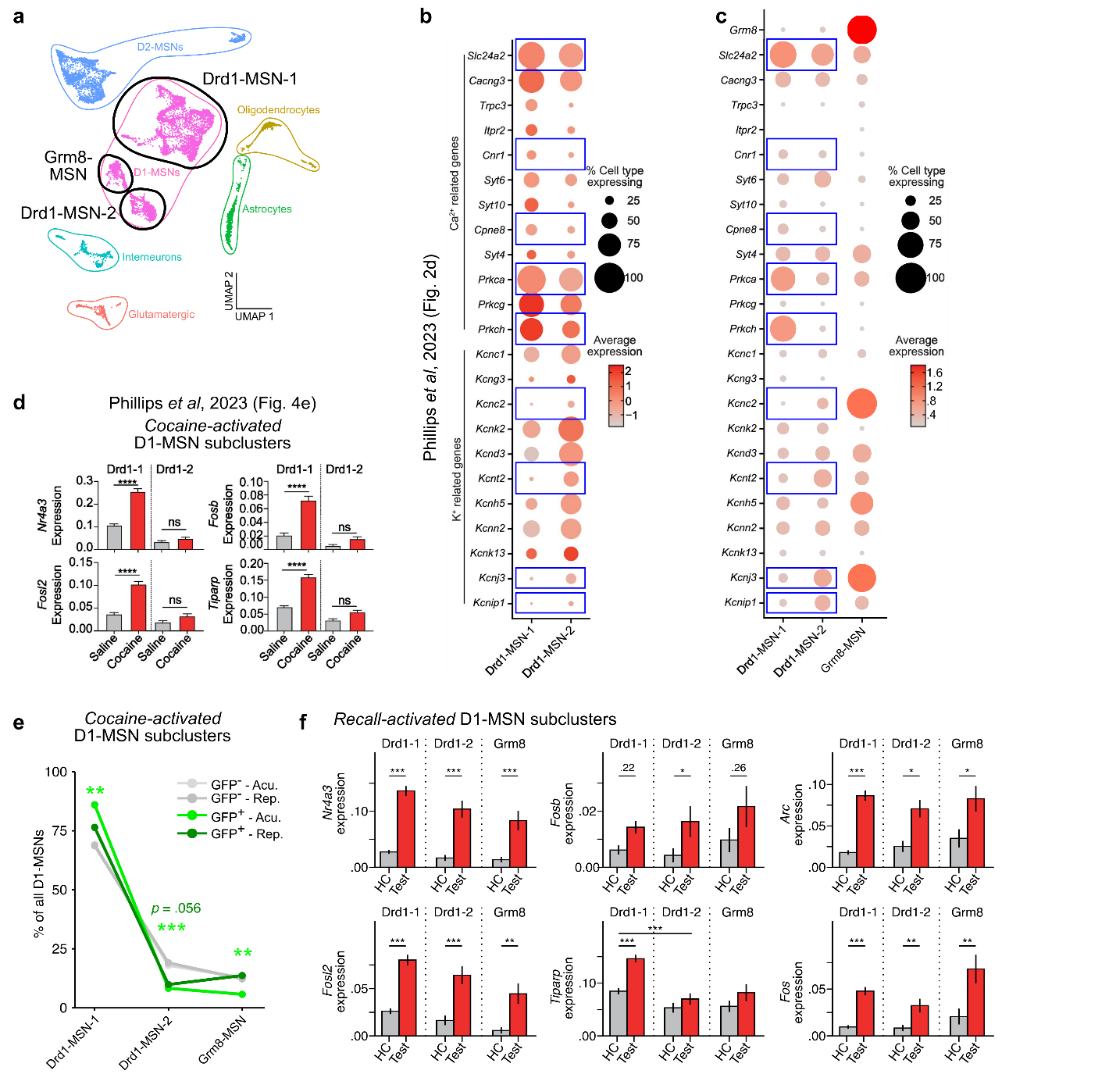
**

**Supplementary Figure 10: Comparison of snRNAseq data with Phillips et al, 2023**^57^**. a**, Subclustering of D1-MSNs into 3 subclusters Drd1-MSN-1, Drd1-MSN-2 and Grm8-MSN, named after Phillips et al, 2023 ^57^. **b**, Differentially expressed genes between Drd1-MSN-1 and Drd1-MSN-2 in Phillips et al, 2023 ^57^. **c**, Differentially expressed genes between Drd1-MSN-1 and Drd1-MSN-2 in the present dataset. Blue boxes indicate qualitative similarity between expression patterns. **d**, Expression of cocaine-activated immediate early gene transcripts between Drd1-MSN-1 and Drd1-MSN-2 subclusters in Phillips et al, 2023 ^57^, showing induction restricted to Drd1-MSN-1. **e**, Proportion of nuclei from each treatment/FANS status combination in D1-MSNs subcluster, highlighting enrichment of GFP+ nuclei (i.e., cocaine-activated nuclei) in Drd1-MSN-1 and depletion in Drd1-MSN-2 as compared to GFP- nuclei. FDR-adjusted *p* values correspond to standardized Pearson’s residuals after χ^2^ tests: Acute: χ^2^ = 76.59, df = 2, ****p* < 0.001. Repeated: χ^2^ = 13.55, df = 2, ***p* = 0.0011. **f**, Expression of immediate early gene transcripts upon CPP memory recall across D1-MSNs subclusters, showing induction in all subclusters except for *Tiparp*. *p*-values were obtained by logistic regression differential testing for these select genes and conditions using *Seurat::FindMarkers* and FDR corrected. *FDR < 0.05, **FDR < 0.01, ***FDR < 0.001. Bar graphs are expressed as means ± SEM. Source data are provided as a Source Data file.

**
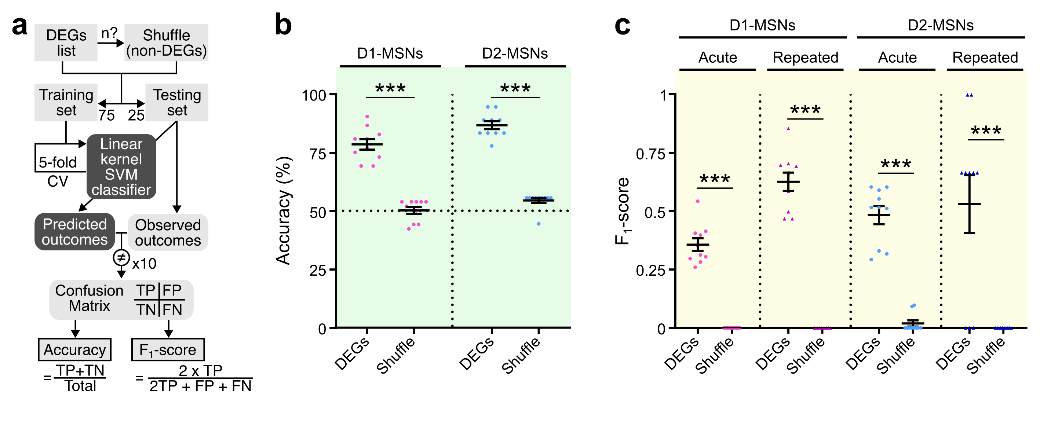
**

**Supplementary Figure 11: Decoding of nuclei identity using DEG information. a**, Conceptual schematic for supervised binary linear classification of nuclei using support vector machines (SVM) with 5-fold cross validation (CV). Features were corresponding DEG lists and outcomes were group membership. For each iteration (10 in total), nuclei were randomly split into training (75%) and testing (25%) sets. As controls for DEGs, a panel of the same size (same number of genes that comprise the corresponding DEG list) was randomly selected among non-DEGs (“Shuffle”). From confusion matrices (TP true positives, FP false positives, TN true negative, FN false negatives), classifier accuracy and F_1_-scores were calculated. **b**, SVM classification of GFP+ nuclei of acute/1P vs repeated/5P origin using Repeated vs Acute DEGs (Fig. 3i) as input features. Two-way ANOVA: interaction cell type x gene list F_1,36_ = 1.38, *p* = 0.2487, main effect of gene list F_1,36_ = 321.9, ****p* < 0.001, main effect of cell type F_1,36_ = 13.65, ****p* = 0.0007; followed by Šidák post-hoc tests. **c**, SVM classification of GFP+ nuclei of acute/1P and repeated/5P origin between Arc+ vs Arc- (reactivation status) using “reactivation” DEGs (Fig. 6d) as input features. Three-way ANOVA: interaction treatment x cell type x gene list F_1,72_ = 2.122 *p* = 0.1495, interaction treatment x cell type F_1,72_ = 3.02 *p* = 0.0864, interaction treatment x gene list F_1,72_ = 5.85 **p* =0.0181, interaction cell type x gene list F_1,72_ = 0.007 *p* = 0.9348, main effect of treatment F_1,72_ = 4.57 **p* = 0.0359, main effect of cell type F_1,72_ = 0.13 *p* = 0.7171, main effect of gene list F_1,72_ = 205.7 *p* < 0.0001; followed by Šidák post-hoc tests. Source data are provided as a Source Data file.
