# Supplementary material for: Cocaine-context memories are transcriptionally encoded in nucleus accumbens Arc ensembles
Source: Nat Commun. 2025 Jul 2;16:6084. doi: 10.1038/s41467-025-61004-9 (PMC12222677; doi:10.1038/s41467-025-61004-9)
Supplement: Supplementary file 2 — Description of Additional Supplementary Files [file 41467_2025_61004_MOESM2_ESM.docx]

**File name: Supplementary Data 1:**

List of primers used for quantitative real-time PCR.

**File name: Supplementary Data 2**

Lists of clusters gene markers.

**File name: Supplementary Data 3**

Lists of DEGs for GFP-positive vs GFP-negative nuclei.

**File name: Supplementary Data 4**

Lists of DEGs for *Arc*-positive vs *Arc*-negative nuclei.

**File name: Supplementary Data 5**

Lists of DEGs for reactivated vs non-reactivated nuclei.

**File name: Supplementary Data 6**

Comparison of DEG lists obtained with different analysis frameworks.
